# ReRNet: A Deep Learning Network for Classifying Blood Cells

**DOI:** 10.1177/15330338231165856

**Published:** 2023-03-28

**Authors:** Ziquan Zhu, Shui-Hua Wang, Yu-Dong Zhang

**Affiliations:** 1 School of Computing and Mathematical Sciences, 4488 University of Leicester, Leicester, UK; 2 School of Computer Science and Technology, Henan Polytechnic University, Jiaozuo, P R China; 3 Department of Information Systems, Faculty of Computing and Information Technology, King Abdulaziz University, Jeddah, Saudi Arabia

**Keywords:** blood cells, ResNet50, randomized neural network, convolutional neural network

## Abstract

**Aims:**

Blood cell classification helps detect various diseases. However, the current classification model of blood cells cannot always get great results. A network that automatically classifies blood cells can provide doctors with data as one of the criteria for diagnosing patients’ disease types and severity. If doctors diagnose blood cells, doctors could spend lots of time on the diagnosis. The diagnosis progress is very tedious. Doctors can make some mistakes when they feel tired. On the other hand, different doctors may have different points on the same patient.

**Methods:**

We propose a ResNet50-based ensemble of randomized neural networks (ReRNet) for blood cell classification. ResNet50 is used as the backbone model for feature extraction. The extracted features are fed to 3 randomized neural networks (RNNs): Schmidt neural network, extreme learning machine, and dRVFL. The outputs of the ReRNet are the ensemble of these 3 RNNs based on the majority voting mechanism. The 5 × 5-fold cross-validation is applied to validate the proposed network.

**Results:**

The average-accuracy, average-sensitivity, average-precision, and average-F1-score are 99.97%, 99.96%, 99.98%, and 99.97%, respectively.

**Conclusions:**

The ReRNet is compared with 4 state-of-the-art methods and achieves the best classification performance. The ReRNet is an effective method for blood cell classification based on these results.

## Introduction

The cells that exist in the blood are called blood cells. It can flow through the whole body through the blood. For mammals, blood cells are roughly divided into platelets, red blood cells, and white blood cells. Transporting oxygen is the main function of red blood cells in the body. White blood cells are mainly responsible for protecting the body. When foreign germs enter the body, it is responsible for destroying these germs. Platelets play a vital part in the hemostasis of the body.

Blood cell classification helps detect various diseases.^1^ The current classification of blood cells is completed by doctors. If doctors manually diagnose blood cells, doctors could spend lots of time on the diagnosis. The diagnosis progress is very tedious. Doctors can make some mistakes when doctors are influenced by some factors, such as feeling tired. On the other hand, different doctors may have different points on the same patient.

A network that automatically classifies blood cells can provide doctors with data as one of the criteria for diagnosing patients’ disease types and severity. More and more researchers proposed a sea of computer models for the classification of blood cells.^2^ Huang et al.^3^ proposed a new network (MGCNN) to classify the blood cell. This network was composed of the convolutional neural network (CNN) and the modulated Gabor wavelet. They calculated the dot product of CNN kernels with multi-scale and orientation Gabor operators. After that, they combined these features to classify the blood cells. Parab and Mehendale^4^ combined image processing technology and CNN for classifying red blood cells. They classified red blood cells into 9 categories. Finally, this method achieved 98.5% accuracy. Lamberti^5^ introduced the SVM model for the classification of red blood cells. This model yielded around 99% accuracy. Liang et al.^6^ presented a combined network (CNN-RNN) to classify blood cells. The custom loss function was selected to speed up the network convergence. When the Xception was selected as the backbone, the network got the best 4-classification accuracy of 90.79%. Banik et al.^7^ designed a fused CNN framework for white blood cell classification. For this framework, there were 5 convolution layers, 3 pooling layers, and one fully connected layer. The experiment showed that this framework could achieve good performance and train faster than the CNN-RNN model. Yang et al.^8^ used Faster R-CNN as the main method to detect cells based on the microscopic images. Lots of experiments’ results showed that this method could save time and get good performance. Imran Razzak and Naz^9^ presented 2 methods for blood smear segmentation and classification. One method was based on the fully conventional network. Another method combined the CNN and extreme machine learning for classifying the blood smear. The segmented method got 98.12% and 98.16% accuracy. The classification accuracy was 94.71% and 98.68%. Choi et al.^10^ applied a dual-stage CNN method for the automatic white blood cell count. The public data set included 2174 images and was divided into 10 classes. The final result achieved 97.13% precision, 97.1% F1, 97.06% recall, and 97.06% accuracy. Sahlol et al.^11^ proposed a new method for classifying white blood cells. The VGG was selected as the backbone of this method and used as the feature extractor. Then, these features were sent to the enhanced Salp Swarm Algorithm. Shahzad et al.^12^ introduced a novel framework (4B-AdditionNet-based CNN) to categorize white blood cells. The contrast-limited adaptive histogram equalization was used for processing. Some CNN networks, ResNet50 and EfficientNetB0, were used as feature extractors. The SVM and quadratic discriminant analysis were used as the classifier. This method got 98.44% accuracy on the blood cell images data set. Loh et al.^13^ proposed a novel computer vision technology (Mask R-CNN) for detecting and segmenting blood cells. This novel technology could save training time and reduce the error. Finally, the Mask R-CNN achieved 94.57% accuracy. Khan et al.^14^ proposed a white blood cells classification network (MLANet-FS). This network selected the AlexNet as the backbone. They exploited different features from different layers of AlexNet to achieve enough details. Then, the extreme learning machine (ELM) was used for white blood cells classification. The accuracy of MLANet-FS was 99.12%. Islam et al.^15^ proposed a new model based on the transformer model for the diagnosis of the malaria parasite. The precision, F1-score, recall, accuracy, and AUC of this new model were 96.99%, 96.44%, 95.88%, 96.41%, and 99.11%. Gavas and Olpadkar^16^ selected 27 CNN models for classifying blood cells. Data augmentation was used in this paper to improve classification performance. Then, the ensemble of the top CNN models was a big improvement. The accuracy of this model was 99.51%. Parayil and Aravinth^17^ offered a model to classify blood cells. Two popular CNN models, DenseNet201 and VGG16, were selected as the feature extractor. The principal component analysis was selected to select features. The result of this method was 89.75% accuracy. Su et al.^18^ offered a novel system to segment white blood cells based on smear images. This novel system was based on CNN models. Three features were selected and then fed to CNN models for segmentation, which were color features, LDP-based texture features, and geometrical features, respectively. There were 450 images for experiments. This system achieved 99.11% accuracy. Harahap et al.^19^ used different CNN models to classify red blood cells. Two different CNN models were selected, which were LeNet-5 and DRNet. By experiments, the LeNet-5 and DRNet could achieve 95% and 97.3% accuracy, respectively. Kousalya et al.^20^ used different CNN models to classify the blood cells. The GoogleNet using LReLU and ReLU can achieve 91.72% and 93.43% accuracy. Chien et al.^21^ combined CNN and Faster R-CNN for the identification and detection of white blood cells. The combined network could get an accuracy of 90%. Lee et al.^22^ offered a new architecture for detecting or counting blood cells. In this architecture, VGG16 was selected as the backbone. The convolutional block attention module, region proposal network, and region of interest pooling were used to improve the classification performance. This architecture achieved 76.1% precision and 95% recall. For the classification and detection of one type of blood cell, Abas and Abdulazeez^23^ proposed 2 different systems, which were named CADM1 and CADM2. These 2 different systems were based on CNN and YOLOv2. By experiments, the CADM2 can achieve 92.4% accuracy and 94% precision. Kumari et al.^24^ designed a novel CNN model to classify white blood cells, which included 2 convolutional layers, 2 max-pooling layers, 2 ReLU layers, 2 fully-connect layers, and softmax. The accuracy of this CNN model was greater than 90%. Girdhar et al.^25^ introduced a new network based on CNN to classify white blood cells. This network was tested on the Kaggle dataset and achieved 98.55% accuracy. Ekiz et al.^26^ used different methods to classify white blood cells. The first method was based on CNN. The other method combined the CNN and SVM model. By comparison, the combination of SVM model and CNN can yield better accuracy, which was 85.96%. Ramadevi et al.^27^ used a CNN model (ConVNet) for the blood cells classification. This framework was tested on 13k images and achieved above 80% accuracy. Ghosh and Kundu^28^ proposed a network which combined the CNN and randomized neural network (RNN). The long short-term memory network was selected to improve the accuracy. The CNN-RNN model achieved 94.13% accuracy for 2-way classification and 87.29% accuracy for 4-way classification. Xiao et al.^29^ introduced a new system for the white blood cell classification. In this system, the EfficientNet was used as the main backbone. Based on this system, it can yield 90% accuracy with high speed.

From the above analysis, many recent methods for classifying blood cells are based on CNN. However, the CNN models have a sea of parameters and layers. It will take a lot of time for training. What's more, the small data set may not have a great classification performance based on the CNN models.

To settle these problems, we offer a novel method (ReRNet) for blood cell classification. In the ReRNet, ResNet50 is selected as the backbone, and 3 RNNs are used for classification. They are SNN, dRVFL, and ELM, respectively. The main contributions of this paper are listed as follows:

- We propose a novel method (ReRNet) for blood cell classification.
- The ResNet50 is validated as the backbone by comparing it with other CNN models.
- The proposed method is compared with other state-of-the-art methods and achieves the best performance.
- Three RNNs are used to improve the classification performance.
- The outputs are the ensemble of the output of 3 RNNs to improve the robustness.

The rest of this paper is as follows: section “Materials” talks about the materials; section “Methodology” is mainly about the methodology used in this paper; the experiments are shown in section “Experiment Results”; the conclusion is presented in section “Conclusion.”

## Materials

The public data set can be available on the Kaggle website (https://www.kaggle.com/datasets/paultimothymooney/blood-cells). This public data set has 4 types of blood cells: neutrophils, monocytes, lymphocytes, and eosinophils. This public data set included 12,500 blood cell images in total. However, some diseases and their complications have no significant effect on neutrophil dynamics.^30^ Therefore, 3 cell types are tested, which are eosinophil, lymphocyte, and monocyte. The number of images of eosinophil, lymphocyte, and monocyte are 3120, 3103, and 3098, respectively.^31^ The details of the data set used in this paper are given in Table 1.

**Table 1.** The Details of the Data Set.

	Eosinophil	Lymphocyte	Monocyte
Training set	2497	2483	2478
Testing set	623	620	620

**Figure 1.**
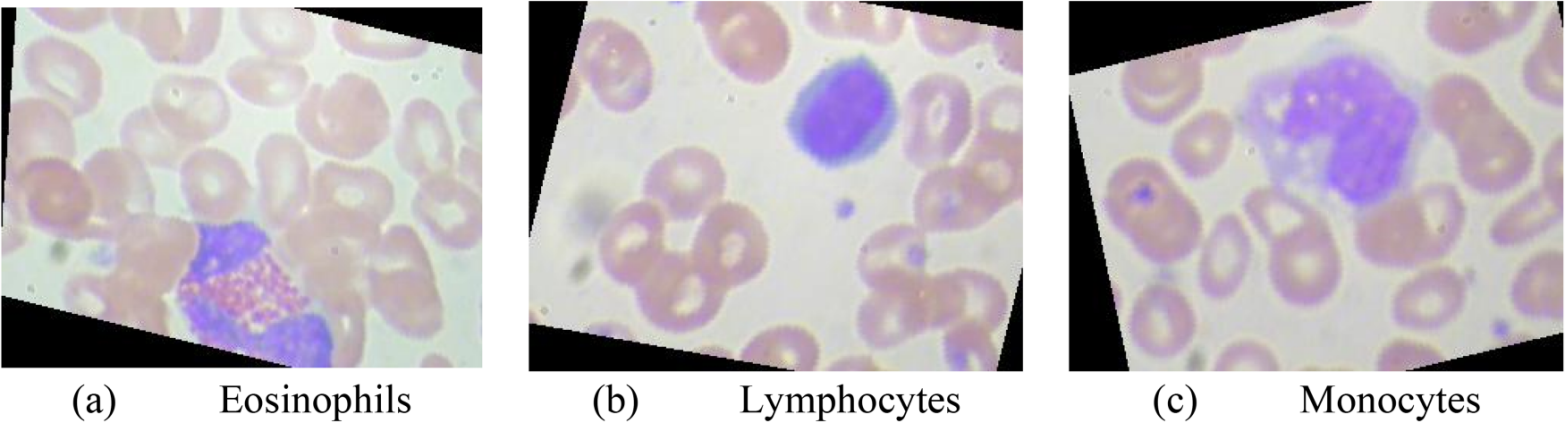
Three classes of blood cells: (a) eosinophils, (b) lymphocytes, (c) monocytes.

Eosinophils account for 0.5% to 3% of the total number of leukocytes. Lymphocytes account for 20% to 30% of the total number of leukocytes, which are round or oval in size. Monocytes account for 3% to 8% of the total number of white blood cells. It is the largest cell in white blood cells. Diameter 14 to 20 μm. The figures of eosinophils, lymphocytes, and monocytes are given in Figure 1.

## Methodology

Computer-aided diagnosis system has been widely used in the medical field based on artificial intelligence and computer vision technology such as medical image detection, segmentation, classification, and so on. The important step in medical image analysis is to extract useful features. With the continuous progress of deep learning technology, the CNN model has become one of the main feature extraction methods in computer-aided diagnosis systems. The function of the convolution layer is to extract features. The pooling layer can reduce the dimension of the feature map to save the calculation amount and time. In the most recent decade, many excellent CNN models have been proposed, such as ResNet,^32^ AlexNet,^33^ VGG,^34^ MobileNetv2,^35^ DenseNet,^36^ UNet,^37^ and so on. The hyperparameters used in this paper are concluded in Table 2.

**Table 2.** The Definition of Hyperparameter.

Hyperparameter	Definition
E(X)	The output of the residual learning
D(X)	The learned feature
($x_{i}$, $h_{i}$)	The given data set
n	The input dimension
m	The output dimension
$b_{j}$	The weights vector
$c_{j}$	The bias of the *j*-th hidden node
d	The final output weight
e	The output bias of SNN
$h=(h_{1},\;\ldots ,h_{N}{)}^{T}$	The ground-truth label matrix of the data set
l	The number of hidden layers in dRVFL
g()	The sigmoid function
v	The number of hidden nodes
M	The output matrix of the hidden layer
T	The input of the output layer for dRVFL
p	The predictions of the three RNNs

### Selection and Modification of Backbone Network

Generally speaking, the more layers of the network, the richer the features of different levels could be extracted. But some experiments show that with the deepening of the network, the optimization effect is worse, and the accuracy is reduced. For example, one is a 56-layers network, and the other is a 20-layers network. Generally, the performance of the 56-layers network should be greater than or equal to the performance of the 20-layers network. However, the error of the 56-layers network may be greater than that of the 20-layers network. The 20-layers network could achieve better classification performance than the 56-layers network.

He et al.^32^ proposed ResNet to solve the above-mentioned problems. For a stacked-layer structure (several layers stacked), the input is represented as *X*, the learned feature is denoted as D(X), and E(X) is got through the residual learning. The formula of the residual learning is shown as:

(1) E(X)=D(X)−X

Based on the above formula, the learned feature can be shown as follows:

(2) D(X)=E(X)+X

**Figure 2.**
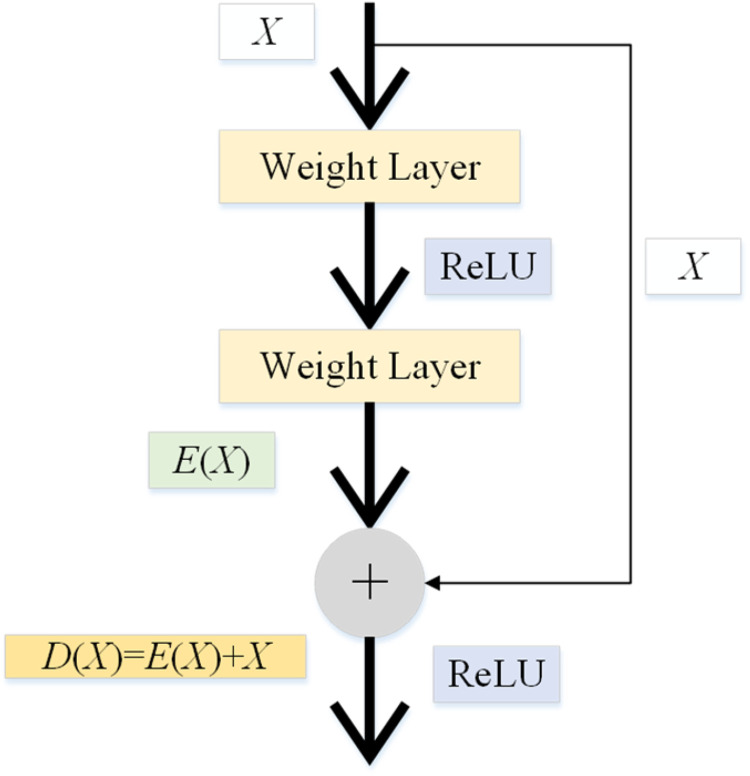
The framework of ResNet.

When the residual is 0, the accumulation layer only performs identity mapping, and the network performance will not be degraded. Therefore, this residual greatly alleviates the degradation problem and enables the accumulation layer to learn new features based on the input features to achieve better performance. The framework of ResNet is given in Figure 2.

**Figure 3.**
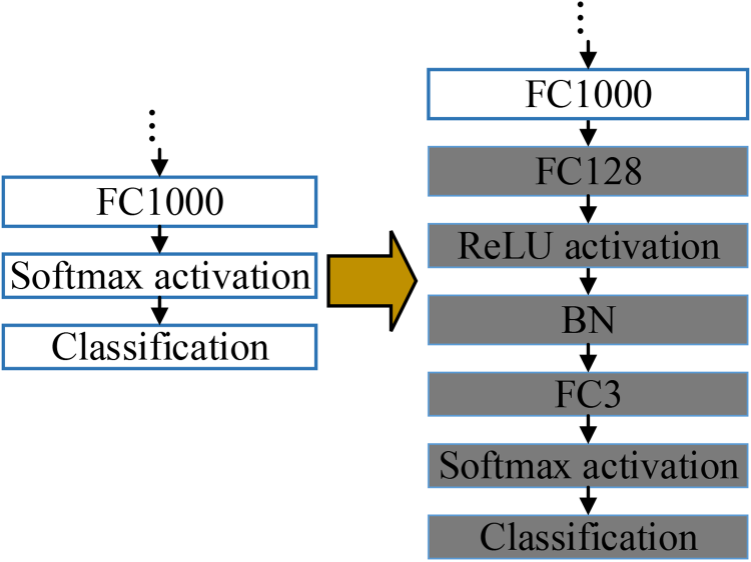
The modifications.

In this paper, transfer learning is used to improve classification performance. Some modifications should be made to the ResNet because of the difference between the ImageNet dataset and the dataset used in this paper, as is demonstrated in Figure 3. The original “Softmax activation” and “Classification” are replaced by the “FC128,” “ReLU,” “BN,” “FC3,” “Softmax activation,” and “Classification,” respectively. The “FC3” is added as there are only 3 categories of images in our dataset. Further, an “FC128” is inserted into the model to mitigate the difference in dimensions between “FC1000” and “FC3.” The modified ResNet is fine-tuned on our dataset, and 3 RNNs replace the last 5 layers to achieve better classification performance. Therefore, the ResNet only serves as the feature extractor in the proposed model, and the “FC128” is the feature layer.

### Proposed Strategy of Ensemble of RNNs

**Figure 4.**
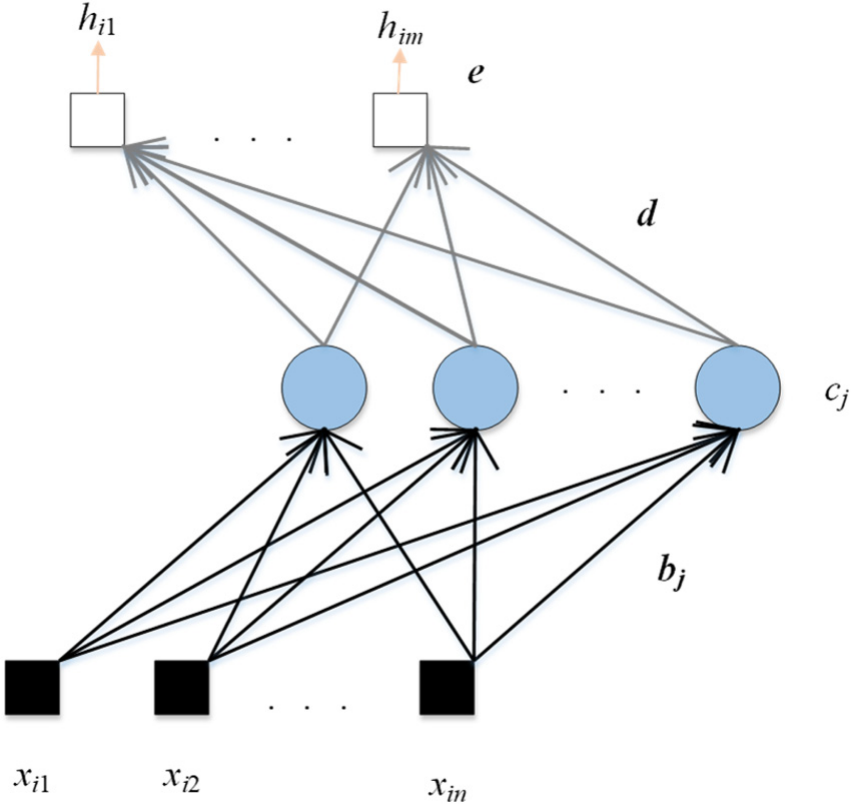
The framework of SNN.

CNN models have achieved amazing results in recent years. For example, they can obtain high accuracy in ImageNet. However, the performance of the CNN model is often unsatisfactory on small data sets. Therefore, we select 3 RNNs in this paper, which are Schmidt neural network (SNN), ELM, and deep random vector function linking (dRVFL). The framework of SNN is shown in Figure 4.

The calculation steps of SNN are given as follows:

(3) $$x_{i}=(x_{i1},\ldots ,x_{in}{)}^{T}\in R^{n},\;i=1,\ldots ,N,$$

(4) $$h_{i}=(Q_{i1},\ldots ,Q_{im}{)}^{T}\in R^{m},i=1,\ldots ,N,$$

where the dataset is set as ($x_{i}$, $h_{i}$), $x_{i}$ is the input, $h_{i}$ is the ground-truth label, *n* and *m* are the dimensions of the input and output.

**Figure 5.**
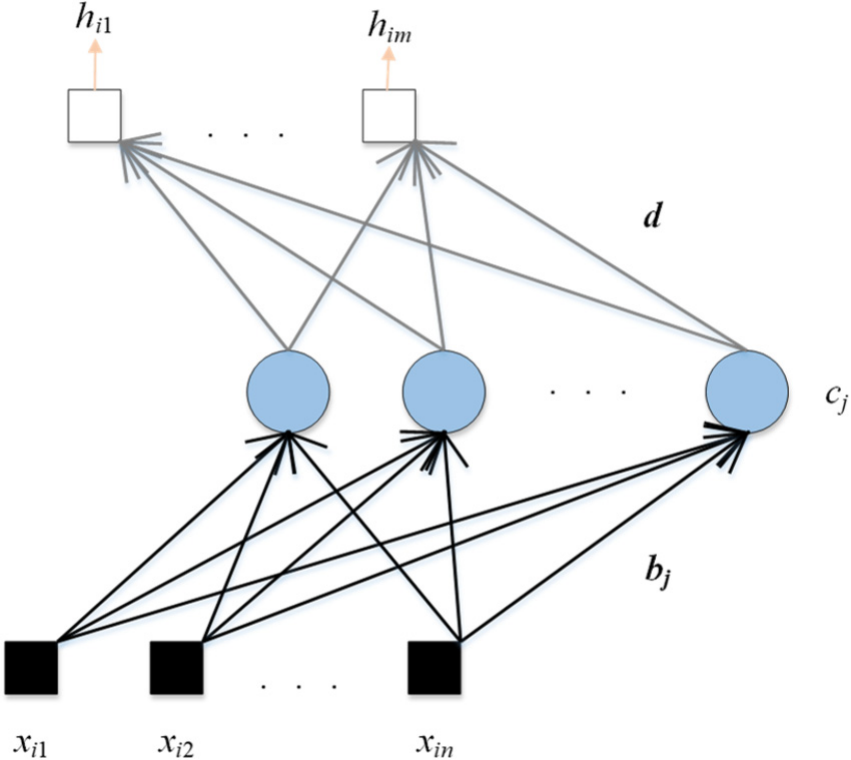
The framework of ELM.

The second calculation step is presented as follows:

(5) $$M_{SNN(i)}=\overset{v}{\underset{\;j=1}{\sum }}\;g(b_{j}x_{i}+c_{j}),\;i=1,\ldots ,N.$$

where g() is the activation function, $b_{j}$ is the weight which connects the input data with the *j*-th hidden node, $c_{j}$ is the bias of the *j*-th hidden node, and *v* represents the number of hidden nodes. After 2 calculation steps, the output weight (d) is calculated as follows:

(6) $$(d,e)=M_{SNN}^{+}h.$$

where e is the bias between the hidden layer and the output layer, the ground-truth label is presented as h. Another RNN used in this paper is ELM, as given in Figure 5.

From the figures of these 2 RNNs, we can see that the only difference between SNN and ELM is that there is an output bias on the SNN. Therefore, for the calculation of ELM, the first 2 steps are the same as SNN. The calculation of output weight (d) for ELM is given as follows:

(7) $$d=M_{ELM}^{+}h.$$

**Figure 6.**
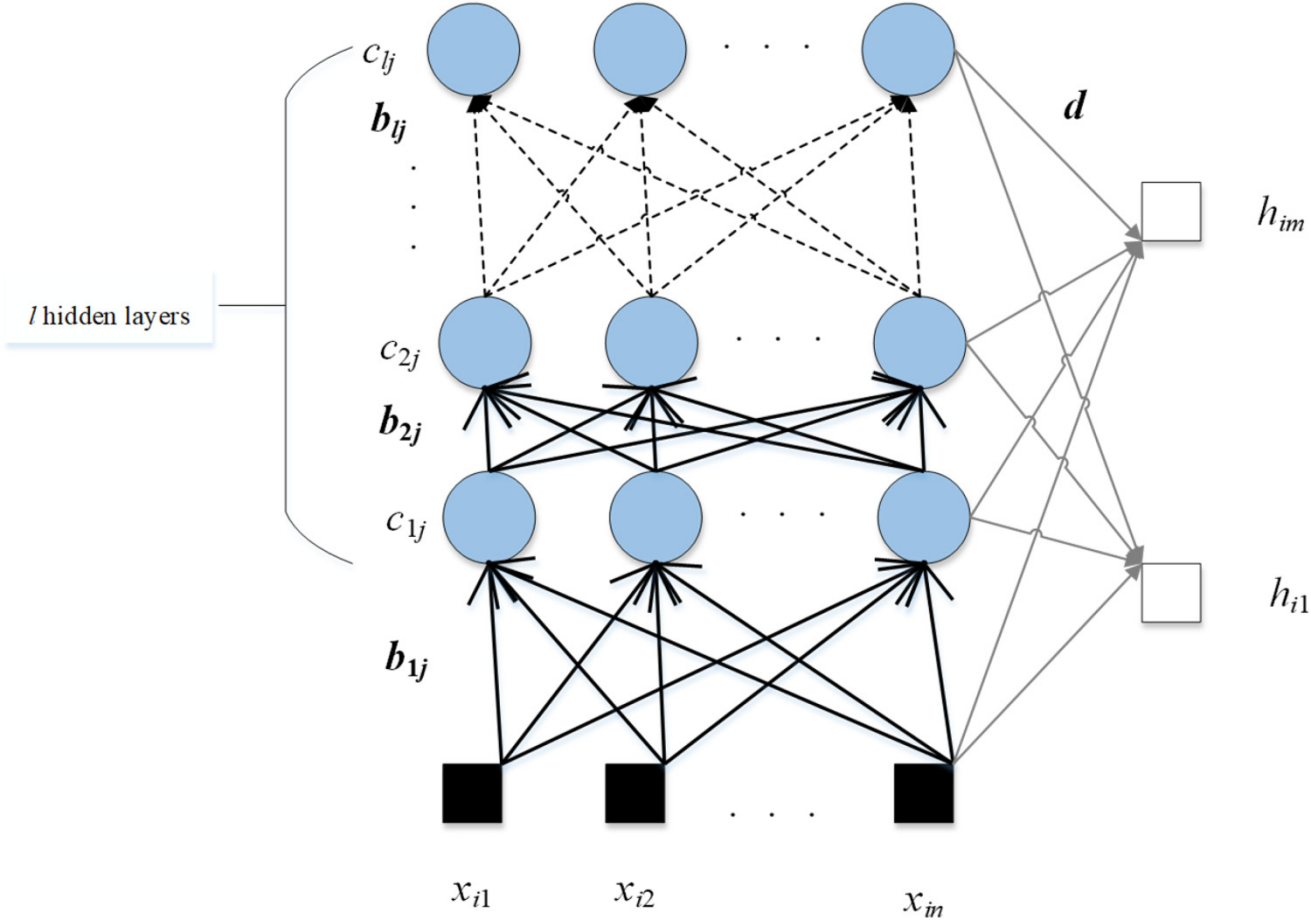
The framework of dRVFL.

As presented in Figure 6, the number of hidden layers is *l*. For SNN and ELM, there is only one hidden layer in their structures. The calculation steps of dRVFL will be a little different.

For the first hidden layer, the calculation is defined as follows:

(8) $$M_{\mathrm{dRVFL}i^{1}}=\overset{v}{\underset{\;j=1}{\sum }}\;g(b_{1j}x_{i}+c_{1j}),\;i=1,\ldots ,N.$$

For other hidden layers, the calculation is presented as follows:

(9) $$M_{\mathrm{dRVFL}i^{l}}=\overset{v}{\underset{\;j=1}{\sum }}\;g(b_{1j}M_{\mathrm{dRVFL}i^{l-1}}+c_{1j}),\;i=1,\ldots ,N.$$

The input of the output layer is shown as follows:

(10) $$T_{\mathrm{RVFL}}=\mathrm{concat}(X,M_{\mathrm{dRVFL}i^{1}},M_{\mathrm{dRVFL}i^{2}},\ldots M_{\mathrm{dRVFL}i^{l}}).$$

The final output weight of dRVFL is given as:

(11) $$d=T_{\mathrm{RVFL}}^{\;+\;}y.$$

The performance of the ensemble of neural networks is usually better than that of the individual network because the ensemble of neural networks is more robust. The parameters in the RNN are random and remain unchanged in the training process. Therefore, bad parameters will lead to poor results. Based on this situation, 3 RNNs are ensembled in this paper, and the error rate can be reduced by majority voting. The formula of the majority voting is given as:

(12) $$\begin{array}{r} U(a_{im})\left\{ \begin{array}{l} p_{t}\;\;\;\;\mathrm{if}\;\exists p_{t}==p_{b},\;t,b\in \{\alpha ,\beta ,\;\gamma \}\\ {\left[1\;0\;0\right]}^{T}\\ \mathrm{otherwise} \end{array}\right.,\;\;\;\;\;\;\;\;\; \end{array}$$

where given the image $a_{im}$, the output function is presented as $U(a_{im})$, the predictions of the 3 RNNs in this paper are $p_{\alpha }$, $p_{\beta }$, and $p_{\gamma }$, respectively, and $[1\;0\;0{]}^{T}$ means the Eosinophil. The output of the proposed model is obtained using the majority voting-based ensemble of the outputs from the 3 RNNs. This paper uses 3 RNNs (SNN, ELM, and dRVFL) for classification, so there will be 3 results. For the majority voting mechanism, when the majority results (more than half of the results) are consistent, the results will be output. When the 3 results are inconsistent, the classification result would be the eosinophil.

### Proposed ReRNet

**Figure 7.**
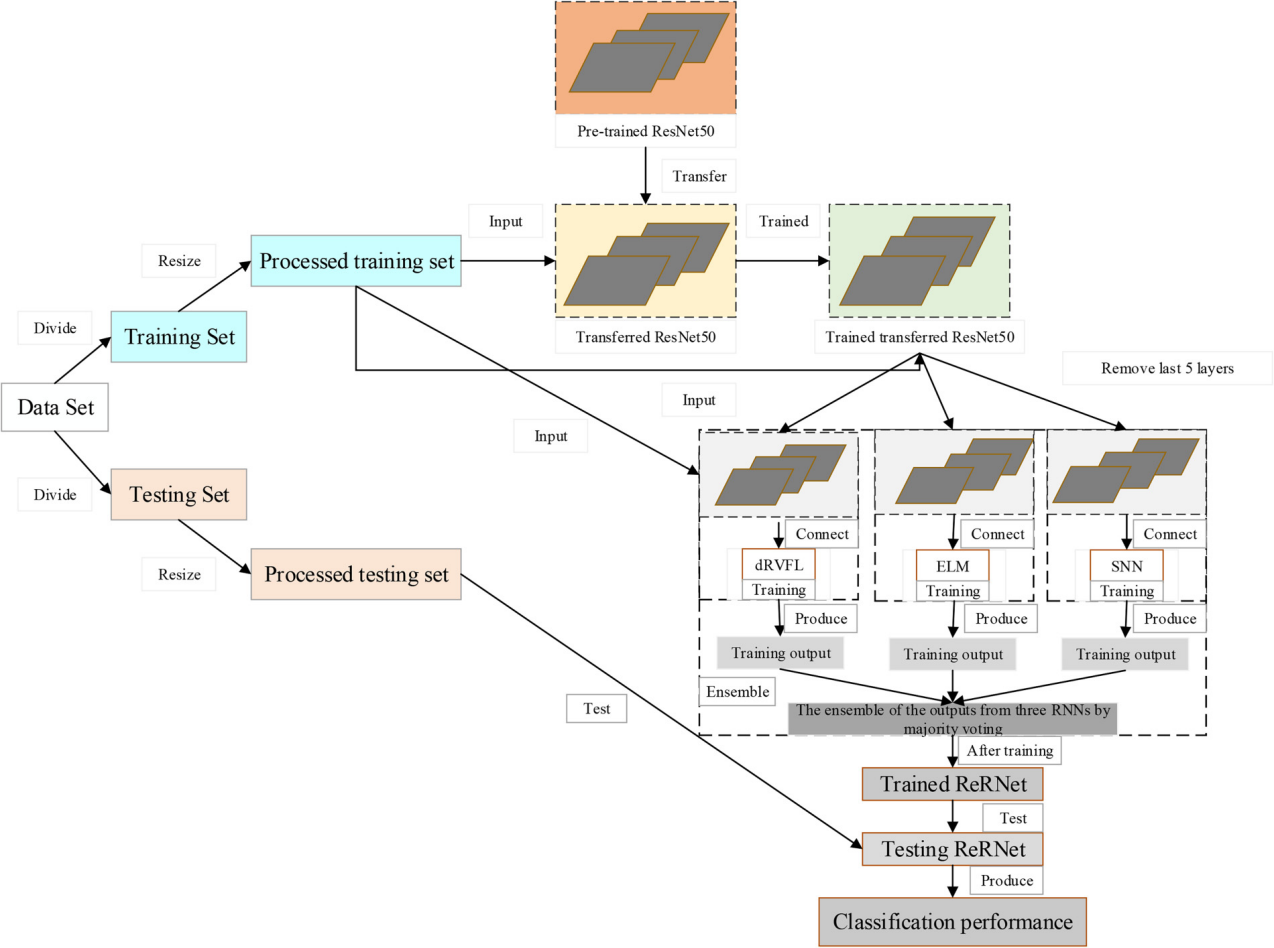
The structure of ReRNet.

We propose a “ResNet50-based ensemble of RNNs (ReRNet)” for blood cell classification. ResNet50 is used as the backbone model for feature extraction. The extracted features are fed to 3 RNNs: SNN, ELM, and dRVFL. The results of the ReRNet are the ensemble of these 3 RNNs based on the majority voting mechanism. The 5 × 5-fold cross-validation is applied to validate the proposed network. The structure of ReRNet is given in Figure 7. The pseudocode of the proposed ReRNet is given in Table 3. Initially, a ResNet50 pre-trained on the ImageNet dataset is selected as the backbone model of the ReRNet. Then, the pre-trained ResNet50 is modified and fine-tuned on the data set used in this paper, and the end 5 layers are replaced by 3 RNNs: SNN, dRVFL, and ELM. So, the ResNet50 can be regarded as the feature extractor in our model. Then, the 3 RNNs are trained with features from the backbone model. Finally, the output of the proposed model is obtained using the majority voting-based ensemble of the outputs from the 3 RNNs.

**Table 3.** The Pseudocode of the Proposed ReRNet.

Step 1: Import the dataset and divide the dataset into training and testing sets.
Step 2: Load the pre-trained ResNet50.
Step 3: Preprocessing Resize samples in the training and testing set based on the input size of ResNet50.
Step 4: Modification of backbone network. Step 4.1 Remove softmax and classification layer. Step 4.2 Add FC128, ReLU, BN, FC3, Softmax activation, and classification.
Step 5: Replace the last five layers with three RNNs.
Step 6: Extract features as the output of the FC128 layer.
Step 7: Train the three RNNs on the extracted features and the labels. Step 7.1: Input is the extracted features. Step 7.2: Target is the images’ labels of the processed training set.
Step 8: Add the majority voting layer. Step 8.1: Ensemble the predictions of the three RNNs Step 8.2: Majority voting of the ensemble of the predictions from the three RNNs. Step 8.3: The whole network is named ReRNet.
Step 9: Test the trained ReRNet on the processed testing set.
Step 10: Report the classification performance of the trained ReRNet.

### Evaluation

**Figure 8.**
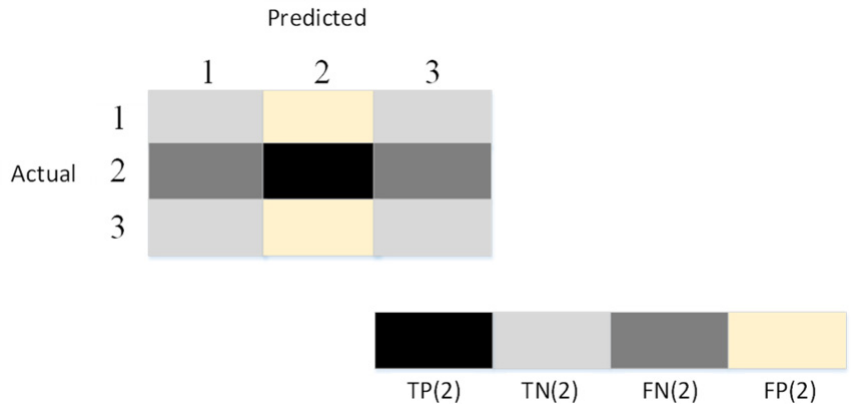
The definitions of TP(2), TN(2), FN(2), and FP(2) when ∂ is 2.

Four multi-classification indexes are used to evaluate the proposed method in this paper: average-accuracy, average-sensitivity, average-precision, and average-F1-score. Because there are 3 classes in this paper, we first define the formulas for accuracy, sensitivity, precision, and F1-score per class as follows:

(13) $$\{\begin{array}{c} \mathrm{accuracy}(\partial )=\frac{\mathrm{TP}(\partial )+\mathrm{TN}(\partial )}{\mathrm{TP}(\partial )+\mathrm{FP}(\partial )+\mathrm{TN}(\partial )+\mathrm{FN}(\partial )}\\ \mathrm{precision}(\partial )=\frac{\mathrm{TP}(\partial )}{\mathrm{TP}(\partial )+\mathrm{FP}(\partial )}\\ \mathrm{sensitivity}(\partial )=\frac{\mathrm{TP}(\partial )}{\mathrm{TP}(\partial )+\mathrm{FN}(\partial )}\\ F10\mathrm{score}(\partial )=\frac{2\times \mathrm{precision}(\partial )\times \mathrm{sensitivity}(\partial )}{\mathrm{precision}(\partial )+\mathrm{sensitivity}(\partial )},\\ \partial =1,\;\ldots ,\;3, \end{array}$$

where ∂ is the number of classes. Three classes are classified in this paper which is a little different from the binary classification. In this situation, 3-class classification can be simplified as 3 binary classifications. When one class is defined as positive, the other 2 classes are defined as negative. For example, when ∂ is 2, the definitions of true positive TP(2), true negative TN(2), false negative FN(2), and false positive FP(2) are presented in Figure 8. The calculations of these 4 multi-classification indexes (average-accuracy, average-sensitivity, average-precision, and average-F1-score) are given as follows:

(14) $$\{\begin{array}{c} \mathrm{average}\text{-}\mathrm{accuracy}=\frac{\sum_{\partial =1}^{3}\;\mathrm{accuracy}(\partial )}{3}\;\\ \mathrm{average}\text{-}\mathrm{precision}=\frac{\sum_{\partial =1}^{3}\;\mathrm{precision}(\partial )}{3}\\ \mathrm{average}\text{-}\mathrm{sensitivity}=\frac{\sum_{\partial =1}^{3}\;\mathrm{sensitivity}(\partial )}{3}\\ \mathrm{average}\text{-}F1\text{-}\mathrm{score}=\frac{\sum_{\partial =1}^{3}\;F1\text{-}\mathrm{score}(\partial )}{3} \end{array},\;\partial =1,\ldots ,3.$$

A confidence interval is an estimated interval for an index. In statistics, the confidence interval of a probability sample is an interval estimate of a certain index. The confidence interval shows the degree to which the true value of the index has a certain probability of falling around the measurement result, which gives the degree of credibility of the measured value of the index. Besides, pairwise comparisons are used to determine which of the 2 models is better for each possible index.

## Experiment Results

### The Settings

The hyper-parameter settings in our ReRNet are demonstrated in Table 4. The mini-batch size is only 10. The max-epoch is set as 2 to avoid overfitting. The learning rate is 1×10^−4^, which is a conventional setting. The only pre-defined hyper-parameter in the 3 RNNs is the number of hidden nodes (v), which is set as 400. The random mapping from lower dimension to higher dimension space is beneficial for the classification.

**Table 4.** The Hyper-Parameter Settings.

Hyper-parameter	Value
Mini-batch size	10
Max-epoch	2
Initial learning rate	1×10^−4^
v	400

Lots of scholars usually would use more max epochs to improve classification performance, such as 30 and 50 max epochs. More max epochs would lead to more training time. Our proposed method gains these results in this paper by only 2 max-epochs for our training, which is much more time-efficient. RNNs (with *v* hidden nodes) with randomly chosen input weights and hidden layer biases can exactly learn *v* distinct observations. Unlike the popular thinking and most practical implementations in that all the parameters of the feedforward networks need to be tuned, one may not necessarily adjust the input weights and first hidden layer biases in applications. Therefore, the RNNs could reduce training time.

### The Performance of ReRNet

The proposed method (ReRNet) is evaluated by 5 × 5-fold cross-validation. We carry out the 5-fold cross-validation by 5 runs to avoid the contingency. The classification performance of these 5 runs is presented in Table 5. The average-accuracy, average-sensitivity, average-precision, and average-F1-score per per-class of each run are given in Table 6. All the evaluations in 5 runs are greater than 99%, proving that the proposed method (ReRNet) is a good choice for classifying blood cells. The running time for 5-fold cross-validation in one run is merely 4786.1 s, which is less time than training deep models because it could take several hours and days to train deep CNN models from scratch.

**Table 5.** The Classification Performance of 5 × 5-Fold Cross-Validation (%).

Run	Fold	Class	Accuracy	Sensitivity	Precision	F1-score
	1	Eosinophil	100.00	100.00	100.00	100.00
		Lymphocyte	100.00	100.00	100.00	100.00
		Monocyte	100.00	100.00	100.00	100.00
	2	Eosinophil	99.95	99.84	100.00	99.92
		Lymphocyte	99.95	100.00	99.84	99.92
		Monocyte	99.95	100.00	100.00	100.00
	3	Eosinophil	100.00	100.00	100.00	100.00
1		Lymphocyte	100.00	100.00	100.00	100.00
		Monocyte	100.00	100.00	100.00	100.00
	4	Eosinophil	100.00	100.00	100.00	100.00
		Lymphocyte	100.00	100.00	100.00	100.00
		Monocyte	100.00	100.00	100.00	100.00
	5	Eosinophil	100.00	100.00	100.00	100.00
		Lymphocyte	100.00	100.00	100.00	100.00
		Monocyte	100.00	100.00	100.00	100.00
	1	Eosinophil	100.00	100.00	100.00	100.00
		Lymphocyte	100.00	100.00	100.00	100.00
		Monocyte	100.00	100.00	100.00	100.00
	2	Eosinophil	99.95	100.00	99.84	99.92
		Lymphocyte	99.95	99.84	100.00	99.92
		Monocyte	99.95	100.00	100.00	100.00
	3	Eosinophil	100.00	100.00	100.00	100.00
2		Lymphocyte	100.00	100.00	100.00	100.00
		Monocyte	100.00	100.00	100.00	100.00
	4	Eosinophil	100.00	100.00	100.00	100.00
		Lymphocyte	100.00	100.00	100.00	100.00
		Monocyte	100.00	100.00	100.00	100.00
	5	Eosinophil	99.95	100.00	100.00	100.00
		Lymphocyte	99.95	100.00	99.84	99.92
		Monocyte	99.95	100.00	99.84	99.92
	1	Eosinophil	100.00	100.00	100.00	100.00
		Lymphocyte	100.00	100.00	100.00	100.00
		Monocyte	100.00	100.00	100.00	100.00
	2	Eosinophil	99.95	100.00	100.00	100.00
		Lymphocyte	99.95	99.84	100.00	99.92
		Monocyte	99.95	99.84	100.00	99.92
	3	Eosinophil	99.95	100.00	100.00	100.00
3		Lymphocyte	99.95	99.84	100.00	99.92
		Monocyte	99.95	99.84	100.00	99.92
	4	Eosinophil	100.00	100.00	100.00	100.00
		Lymphocyte	100.00	100.00	100.00	100.00
		Monocyte	100.00	100.00	100.00	100.00
	5	Eosinophil	99.89	99.84	99.84	99.84
		Lymphocyte	99.89	99.84	99.84	99.84
		Monocyte	99.89	100.00	100.00	100.00
	1	Eosinophil	100.00	100.00	100.00	100.00
		Lymphocyte	100.00	100.00	100.00	100.00
		Monocyte	100.00	100.00	100.00	100.00
	2	Eosinophil	99.89	100.00	100.00	100.00
		Lymphocyte	99.89	99.68	100.00	99.84
		Monocyte	99.89	99.68	100.00	99.84
	3	Eosinophil	100.00	100.00	100.00	100.00
4		Lymphocyte	100.00	100.00	100.00	100.00
		Monocyte	100.00	100.00	100.00	100.00
	4	Eosinophil	99.89	100.00	100.00	100.00
		Lymphocyte	99.89	99.84	99.84	99.84
		Monocyte	99.89	99.84	99.84	99.84
	5	Eosinophil	100.00	100.00	100.00	100.00
		Lymphocyte	100.00	100.00	100.00	100.00
		Monocyte	100.00	100.00	100.00	100.00
	1	Eosinophil	100.00	100.00	100.00	100.00
		Lymphocyte	100.00	100.00	100.00	100.00
		Monocyte	100.00	100.00	100.00	100.00
	2	Eosinophil	100.00	100.00	100.00	100.00
		Lymphocyte	100.00	100.00	100.00	100.00
		Monocyte	100.00	100.00	100.00	100.00
	3	Eosinophil	100.00	100.00	100.00	100.00
5		Lymphocyte	100.00	100.00	100.00	100.00
		Monocyte	100.00	100.00	100.00	100.00
	4	Eosinophil	100.00	100.00	100.00	100.00
		Lymphocyte	100.00	100.00	100.00	100.00
		Monocyte	100.00	100.00	100.00	100.00
	5	Eosinophil	99.89	100.00	99.84	99.92
		Lymphocyte	99.89	99.68	100.00	99.84
		Monocyte	99.89	99.84	100.00	99.92

**Table 6.** The Per-Class Performance of 5 Runs (Average of the 5 Folds).

Run	Class	Average-accuracy	Average-sensitivity	Average-precision	Average-F1-score
	Eosinophil	99.99	99.97	100.00	99.98
1	Lymphocyte	99.99	100.00	99.97	99.98
	Monocyte	99.99	100.00	100.00	100.00
	Eosinophil	99.98	100.00	99.97	99.98
2	Lymphocyte	99.98	99.97	99.97	99.97
	Monocyte	99.98	100.00	99.97	99.98
	Eosinophil	99.96	99.97	99.97	99.97
3	Lymphocyte	99.96	99.90	99.97	99.94
	Monocyte	99.96	99.94	100.00	99.97
	Eosinophil	99.96	100.00	100.00	100.00
4	Lymphocyte	99.96	99.90	99.97	99.94
	Monocyte	99.96	99.90	99.97	99.94
	Eosinophil	99.98	100.00	99.97	99.98
5	Lymphocyte	99.98	99.94	100.00	99.97
	Monocyte	99.98	99.97	100.00	99.98

### Effects of Different Backbones

In this paper, we test 5 different pre-trained CNN models as the backbones: AlexNet, MobileNet, ResNet18, ResNet50, and VGG, respectively. The results of these 5 pre-trained CNN models are shown in Table 7. From the comparison of results, we can find that the ReRNet achieves the best results. We can conclude that the proposed method with ResNet50 as the backbone is an effective tool for blood cell classification.

**Table 7.** The Results of These 4 Pre-Trained CNN Models (%).

Model	Average-accuracy	Average-sensitivity	Average-precision	Average-F1-score
AlexNet	86.28	89.45	83.05	89.62
MobileNet	99.75	99.61	99.89	99.75
ResNet18	99.89	99.8	99.93	99.89
VGG	62.80	67.51	63.12	61.36
**ReRNet (ours)**	**99**.**97**	**99**.**96**	**99**.**98**	**99**.**97**

Bold means the best results.

### Effects of Ensemble of RNNs

We compare the proposed method (ReRNet) with 3 individual RNNs to verify the superiority of the RNNs ensemble. The statistics of 3 RNNs in 5 experiments for blood cells are presented in Table 8. The results of the proposed method are better than the other 3 methods. RNN is recognized as an unstable network because of the randomly fixed parameters during training. The ensemble of 3 individual RNNs could improve the classification performance. In conclusion, the ensemble of RNNs can improve classification performance.

**Table 8.** The Statistics of 3 RNNs in 5 Runs (%).

Run	Model	Average-accuracy	Average-sensitivity	Average-precision	Average-F1-score
1	ResNet50-SNN	99.84	99.79	99.90	99.84
	ResNet50-ELM	99.86	99.86	99.86	99.86
	ResNet50-dRVFL	99.95	99.94	99.96	99.95
	**ReRNet (ours)**	**99**.**99**	**99**.**99**	**99**.**99**	**99**.**99**
2	Model	Average-accuracy	Average-sensitivity	Average-precision	Average-F1-score
	ResNet50-SNN	99.86	99.84	99.88	99.86
	ResNet50-ELM	99.95	99.94	99.98	99.96
	ResNet50-dRVFL	99.95	99.95	99.95	99.95
	**ReRNet (ours)**	**99**.**98**	**99**.**99**	**99**.**97**	**99**.**98**
3	Model	Average-accuracy	Average-sensitivity	Average-precision	Average-F1-score
	ResNet50-SNN	99.89	99.84	99.95	99.89
	ResNet50-ELM	99.89	99.85	99.93	99.89
	ResNet50-dRVFL	99.95	99.91	99.98	99.95
	**ReRNet (ours)**	**99**.**96**	**99**.**94**	**99**.**98**	**99**.**96**
4	Model	Average-accuracy	Average-sensitivity	Average-precision	Average-F1-score
	ResNet50-SNN	99.86	99.80	99.92	99.86
	ResNet50-ELM	99.92	99.94	99.91	99.92
	ResNet50-dRVFL	99.95	99.94	99.96	99.95
	**ReRNet (ours)**	**99**.**96**	**99**.**93**	**99**.**98**	**99**.**96**
5	Model	Average-accuracy	Average-sensitivity	Average-precision	Average-F1-score
	ResNet50-SNN	99.84	99.82	99.86	99.84
	ResNet50-ELM	99.94	99.91	99.96	99.94
	ResNet50-dRVFL	99.95	99.91	99.98	99.95
	**ReRNet (ours)**	**99**.**98**	**99**.**97**	**99**.**99**	**99**.**98**

Bold means ourresults.

**Figure 9.**
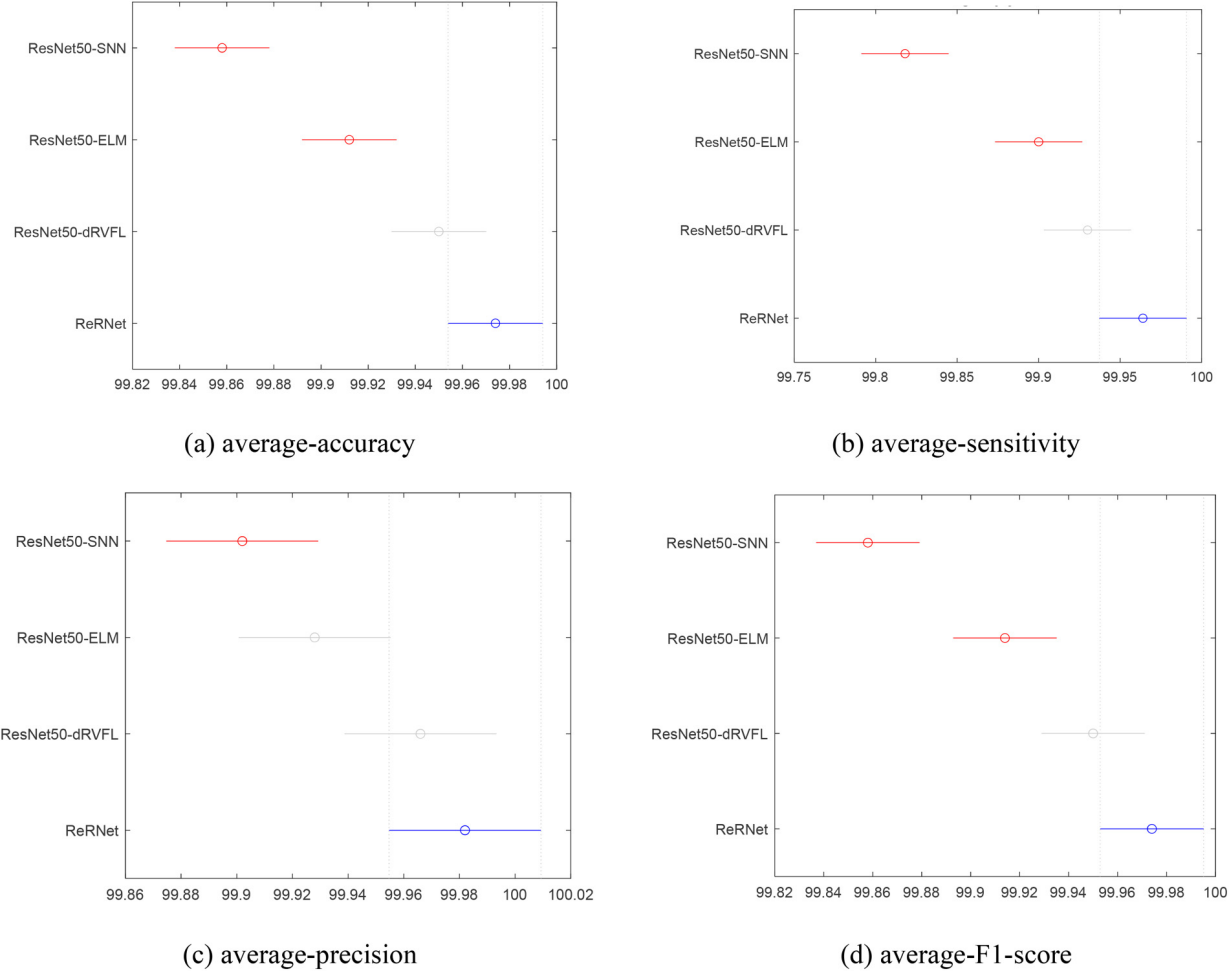
The pairwise comparison of 4 indexes*.* (a) average-accuracy, (b) average-sensitivity, (c) average-precision, (d) average-F1-score.

We also conduct the pairwise comparison based on the results of Table 8. The pairwise comparison of 4 indexes is presented in Figure 9. The proposed model's average-accuracy, average-sensitivity, and average-F1-score are better than ResNet50-SNN and ResNet50-ELM and at the same level as ResNet50-dRVFL. The average-precision of ResNet50-ELM and ResNet50-dRVFL is close to the proposed model. In conclusion, ReRNet achieves good performance, even though the difference between ResNet50-dRVFL and ReRNet is marginal.

### Comparison With the Backbone

The proposed method (ReRNet) is compared with transferred ResNet50 to verify the superiority of the proposed method. The results of the comparison are given in Table 9. The result of our ReRNet is obtained by averaging 5 runs. Based on Table 9, we can conclude that all the results gained from the proposed method are more excellent than those from transferred ResNet50.

**Table 9.** The Results of Comparison With the Backbone.

	Average-accuracy	Average-sensitivity	Average-precision	Average-F1-score
Transferred ResNet50	98.48	98.36	98.59	98.48
**ReRNet (ours)**	**99**.**97**	**99**.**96**	**99**.**98**	**99**.**97**

Bold means the best results.

### Explainability of the Proposed ReRNet

**Figure 10.**
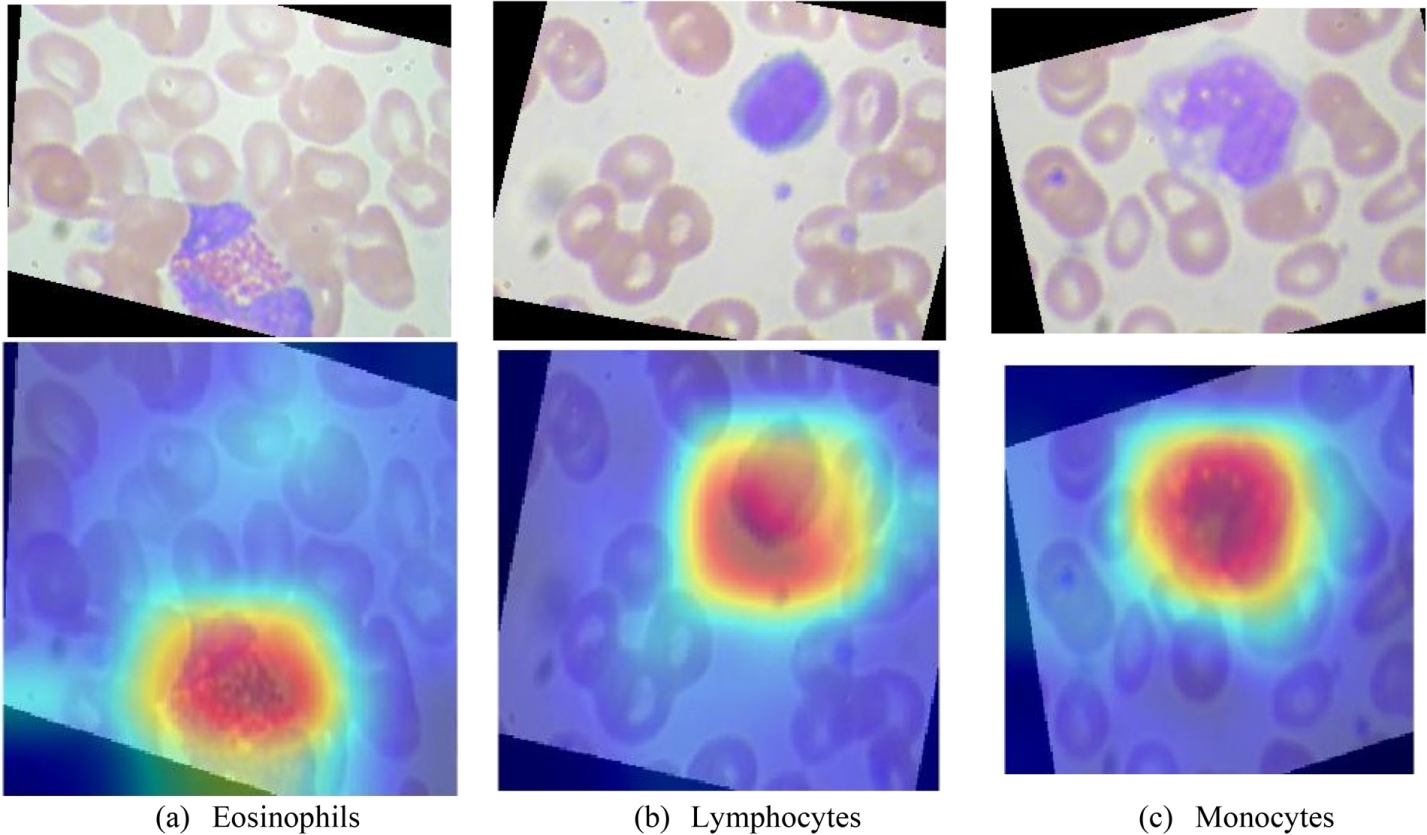
Explainability of the ReRNet: (a) eosinophils, (b) lymphocytes, (c) monocytes.

CNN is just like the black box in applications. Therefore, it is very significant to explain CNN. In this paper, we select gradient-weighted class activation mapping (Grad-CAM) to explain the proposed method (ReRNet) and to figure out how the proposed method makes predictions. We can visualize the attention of ReRNet based on the Grad-CAM. The figures of Grad-CAM are presented in Figure 10. The red regions are the attention of the proposed method. The proposed method would pay less attention to the blue regions. It can be concluded that the ReRNet has the ability to capture blood cells.

### Comparison With Other State-of-the-art Methods

This paper compares our proposed method (ReRNet) with other state-of-the-art methods for blood cell classification. Four state-of-the-art methods are selected to compare with the proposed method, which is the SVM polynomial model,^5^ CNN-RNN,^6^ Fused CNN,^7^ and 4B-AdditionNet,^12^ respectively. The results are presented in Table 10. We can see that our ReRNet achieved the best results, proving our method effectively classifies blood cells.

**Table 10.** The Comparison With Other State-of-the-art Methods (%).

Method	Average-accuracy	Average-sensitivity	Average-precision	Average-F1-score
SVM polynomial^5^	98.1	—	—	—
CNN-RNN^6^	90.79	—	—	—
Fused CNN^7^	95.19	—	92.32	—
4B-AdditionNet^12^	98.44	97.80	98.87	97.23
**ReRNet (ours)**	**99**.**97**	**99.96**	**99.98**	**99.97**

Bold means the best results.

### The Generality of the Proposed Model

To prevent the significant overfitting problem and verify the generality of the proposed model, we test our model on another blood cell data set named malaria cell dataset, which is public and available on the Kaggle website (https://www.kaggle.com/datasets/iarunava/cell-images-for-detecting-malaria). We compare our model with other state-of-the-art methods on this public data set. The comparison results are presented in Table 11. The comparison results show that our models can achieve the best results with other state-of-the-art methods. In conclusion, our model can yield good results in other public data sets.

**Table 11.** The Comparison With Other State-of-the-art Methods on Malaria Cell Dataset (%).

Method	Average-accuracy	Average-sensitivity	Average-precision	Average-F1-score
Customized CNN^38^	93.46	92.59	94.33	—
DeepMCNN^39^	—	92.00	90.00	—
Computer-automated-CNN^40^	—	91.60	94.10	—
ROENet^41^	95.32	94.30	96.34	95.27
**ReRNet (ours)**	**96.45**	**95**.**73**	**97**.**15**	**96.43**

Bold means the best results.

## Conclusion

In this paper, we propose a novel method (ReRNet) for classifying blood cells. The ResNet50 is selected as the backbone of this novel method. Three RNNs (SNN, dRVFL, and ELM) are used for classification. The results of the proposed method are generated by the ensemble of results of 3 RNNs by majority voting. The average-accuracy, average-sensitivity, average-precision, and average-F1-score are 99.97%, 99.96%, 99.98%, and 99.97%. It proves that the proposed method is effective for blood cell classification.

In future research, we will apply this method to other data sets. What’s more, it is very significant for segmenting blood cells. Therefore, we will pay more attention to the segmentation of blood cells. More methods will be tested for blood cell classification, such as UNet, transformer, etc.

## Abbreviations

CNN: convolutional neural network
dRVFL: deep random vector function linking
ELM: extreme learning machine
ReRNet: ResNet50-based ensemble of RNNs
RNN: random neural network
SNN: Schmidt neural network
